# On the existence of a generalized non-specific task-dependent network

**DOI:** 10.3389/fnhum.2015.00430

**Published:** 2015-08-06

**Authors:** Kenneth Hugdahl, Marcus E. Raichle, Anish Mitra, Karsten Specht

**Affiliations:** ^1^Department of Biological and Medical Psychology, University of BergenBergen, Norway; ^2^Division of Psychiatry, Haukeland University Hospital, BergenNorway; ^3^Department of Radiology, Haukeland University Hospital, BergenNorway; ^4^NORMENT Center of Excellence, University of BergenBergen, Norway; ^5^Department of Radiology, Washington University School of Medicine, St. Louis, MIUSA; ^6^Department of Clinical Engineering, Haukeland University Hospital, BergenNorway

**Keywords:** extrinsic mode network (EMN), fMRI, cortical networks, connectivity, cognition, problem solving, default mode network (DMN)

## Abstract

In this paper we suggest the existence of a generalized task-related cortical network that is up-regulated whenever the task to be performed requires the allocation of generalized non-specific cognitive resources, independent of the specifics of the task to be performed. We have labeled this general purpose network, the extrinsic mode network (EMN) as complementary to the default mode network (DMN), such that the EMN is down-regulated during periods of task-absence, when the DMN is up-regulated, and vice versa. We conceptualize the EMN as a cortical network for extrinsic neuronal activity, similar to the DMN as being a cortical network for intrinsic neuronal activity. The EMN has essentially a fronto-temporo-parietal spatial distribution, including the inferior and middle frontal gyri, inferior parietal lobule, supplementary motor area, inferior temporal gyrus. We hypothesize that this network is always active regardless of the cognitive task being performed. We further suggest that failure of network up- and down-regulation dynamics may provide neuronal underpinnings for cognitive impairments seen in many mental disorders, such as, e.g., schizophrenia. We start by describing a common observation in functional imaging, the close overlap in fronto-parietal activations in healthy individuals to tasks that denote very different cognitive processes. We now suggest that this is because the brain utilizes the EMN network as a generalized response to tasks that exceeds a cognitive demand threshold and/or requires the processing of novel information. We further discuss how the EMN is related to the DMN, and how a network for extrinsic activity is related to a network for intrinsic activity. Finally, we discuss whether the EMN and DMN networks interact in a common single brain system, rather than being two separate and independent brain systems.

## An Incidental Observation – Similar Brain Activations, Different Cognitive Tasks

**FIGURE 1:**
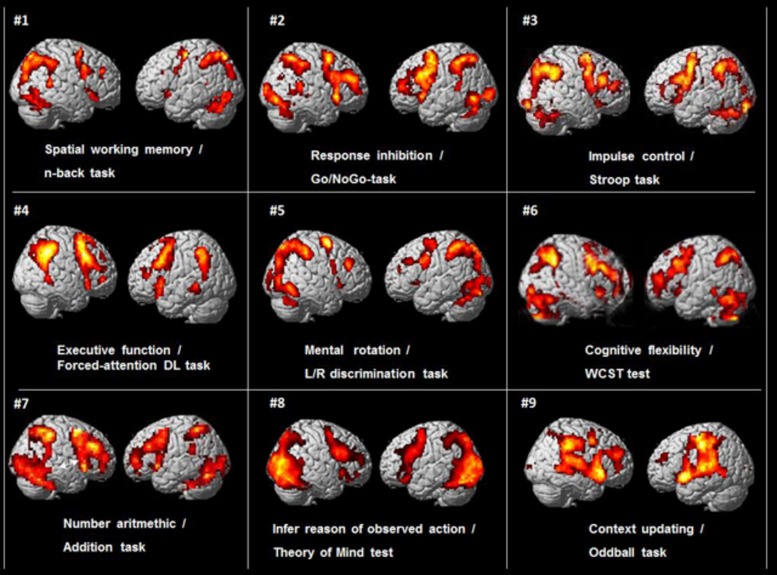
**BOLD-functional magnetic resonance imaging (fMRI) activations from nine different cognitive studies by the Bergen fMRI Group (panels #1–9), with nine different cognitive tasks, visualized on standard SPM templates.** There were different subject groups in the nine different studies.

The origin of this paper was an incidental observation when preparing for a lecture by one of the authors (KH), that when comparing brain activation patterns from functional magnetic resonance imaging (fMRI) data across different studies done in our laboratory at the Bergen fMRI Group, University of Bergen, Norway over the last 10–15 years, a common pattern of activation emerged despite that these studies had used different cognitive stimulus paradigms and tasks. This is shown in **Figure 1**.

As can be seen in **Figure 1** there is a commonality of activations that involves the pre-central, middle and inferior frontal gyri, the intraparietal sulcus and inferior parietal lobule, the inferior posterior temporal region, occipital cortex. In addition, these different tasks typically also engage the dorsal part of the anterior and middle cingulate cortex (ACC) and the supplementary motor area (SMA) medially (not seen in **Figure 1**, but see **Figure 5**). The activations are seen in both hemispheres, but are more prominent in the left hemisphere. The initial reaction when seeing this pattern was that something must have gone wrong in the collection of data when preparing for the seminar lecture.

## Subjects, Cognitive Functions and Tasks in the Studies in **Figure 1**

The subjects in the studies presented in **Figure 1** were healthy adults between 18 and 40 years, with a total of 187 subjects, 117 females, and 70 males. The subject-groups were non-overlapping between studies, i.e., there were different subjects in all the studies in **Figure 1**. The activations were from contrasts for the “hard condition,” following the terminology used by Duncan (2013) and Fedorenko et al. (2013), i.e., using the cognitively most demanding condition when the design involved several conditions that differed in cognitive load or demands. For example, the activation pattern shown for the Go/NoGo task (panel #2 in **Figure 1**) involved pressing the response key whenever a red traffic light was presented in the LCD goggles the subjects wore (Go), and withholding the response whenever a green traffic light was presented (NoGo), i.e., a “hard condition” since green always means “go” and red always means “stop” in everyday life, and the opposite condition will therefore be more cognitively demanding. Correspondingly, the activation pattern seen for the Stroop task (panel #3 in **Figure 1**) was for the “hard condition” of deciding whether the color of the ink of the word matched the color seen before, i.e., the “hard condition” of ignoring the interfering semantic information. The activations in **Figure 1** are for the contrasts between task- processing epochs when the stimulus was present minus passive, resting epochs when the stimulus was absent, except for in the two event-related designs where activations for stimulus targets were contrasted against non-targets. Further details about number of subjects, males, females, age-range, type of MR scanner, analysis software, experimental design, and *p*-thresholds are presented in **Table 1**.

**Table 1:** Overview of experimental task used, cognitive process studied, number of subjects, split for males and females, age, type of MR scanner, analysis software, fMRI design, and p-significance threshold for activated areas, for the nine studies shown in **Figure 1**.

Task/Test	# Subjects (M/F)	Age (years)	MR scanner	Analysis software	Design	*p*-threshold
2-back	26 (12/14)	21 -27	GE Signa 3T	SPM2	Block	0.001/Uncorr
Go-NoGo	13 (13/0)	24–32	Siemens 1.5T	SPM2	Mixed	0.05/FWE
Stroop	16 (8/8)	19–31	GE Signa 3T	SPM2	Block	0.05/FDR
Forced-attention DL	40 (20/20)	22–30	GE Signa 3T	SPM8	Event-related	0.05/FWE
Left/right discrimination	31 (16/15)	18–28	GE Signa 3T	SPM8	Block	0.05/FWE
WCST	14 (14/0)	20–30	Siemens 1.5T	SPM2	Block	0.01/FDR
Addition task	12 (7/5)	25–31	Siemens 1.5T	SPM2	Block	0.05/FWE
Theory of Mind	20 (20/0)	20-30	GE Signa 3T	SPM8	Block	0.05/FWE
Auditory oddball	15 (7/8)	21–28	Siemens 1.5T	SPM2	Event-related	0.05/FWE
Sum total	187 (117/70)					

*FWE, Family Wise Error correction; FDR, False Discovery Rate correction; Uncorr, Uncorrected.*

The different cognitive functions and tasks used in the nine different studies from the Bergen fMRI Group laboratory and shown in **Figure 1** were; Spatial working memory, *n*-back task (panel #1), decide if a number presented in one of nine squares shown in goggles was the same location as the presentation two numbers back (Lycke et al., 2008). Response inhibition, Go/NoGo-task (panel #2), traffic-light pole was presented in the goggles with red or green light, subject had to press a button whenever the green (or red) light was presented and withhold the response whenever the red (green) light was presented (Gundersen et al., 2008). Impulse control, Stroop color–words task (panel #3), decide the color of the ink of color-words when there was a conflict with the semantic meaning of the word, e.g., the word red written in blue ink (Viken, 2007). Executive function, forced-attention dichotic listening task (panel #4), correctly identify simple speech sounds presented through headphones in the left ear in a dichotic listening task, with a conflicting sound simultaneously presented in the right ear (Falkenberg et al., 2011). Mental rotation, left/right discrimination task (panel #5), decide if the fingers of two rotated hands shown in the LCD goggles were of the same or different hand (Hjelmervik et al., 2015). Cognitive flexibility, Wisconsin Card Sorting Test (WCST) (panel # 6), find the solution to pre-set rules for the sorting of cards presented in the goggles (Specht et al., 2009). Arithmetic task (panel #7), decide if two consecutive numbers shown in the goggles add up to a pre-specified sum (Hugdahl et al., 2004). Beliefs about others, Theory of Mind test (panel #8), infer the reason of an observed action (Specht et al., in preparation). Context updating, oddball detection task (panel #9), press a button whenever heard a tone with a deviating pitch in a stream of standard tones with the same pitch (Eichele et al., 2005). All tasks were standard cognitive or neuropsychology tasks or tests that had been adapted to the MR scanner environment, with a slight modification of the Stroop task that also had a working memory component. All tasks had about the same duration and involved either a motor or a verbal response, with approximately the same frequency of responses across tasks.

## Conjunction Analysis – Areas Jointly Activated and Deactivated

Although these tasks denote different cognitive processes and functions; working memory (*n*-back task), response inhibition (Go/No-Go task), impulse control (Stroop color–words task), executive function (dichotic listening task), mental rotation (left–right, L/R, discrimination task), cognitive flexibility (WCST), attribution of beliefs to others (Theory of Mind test), mental arithmetic (addition task), context updating (auditory oddball task), there is also a commonality across tasks; they all require allocation of intellectual capacity for correctly responding to the challenges posed by these tasks.

### Conjunction Analysis across the Studies in **Figure 1**

**FIGURE 2:**
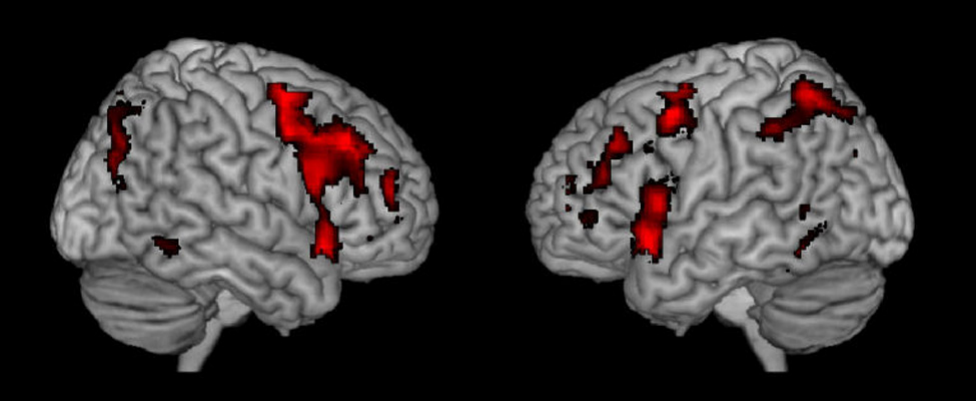
**Resulting joint activations after conjunction analysis of the nine different studies shown in Figure 1**.

To further probe the commonalities of activations across task and cognitive processes, we performed a conjunction analysis across studies and data sets. The conjunction analysis was done by converting the spmT maps from the nine respective studies into *Z*-maps that are independent from degrees of freedom. Inclusive conjunction was estimated as a global conjunction analysis and thus based on a minimal *Z*-value statistics and a cumulative *p*-value of *p* < 0.0001 was applied, corresponding to a threshold for the single studies as the ninth root of 0.0001. The result is seen in **Figure 2** and supports the findings shown in **Figure 1** from observing the different activations across studies when they are displayed together.

**FIGURE 3:**
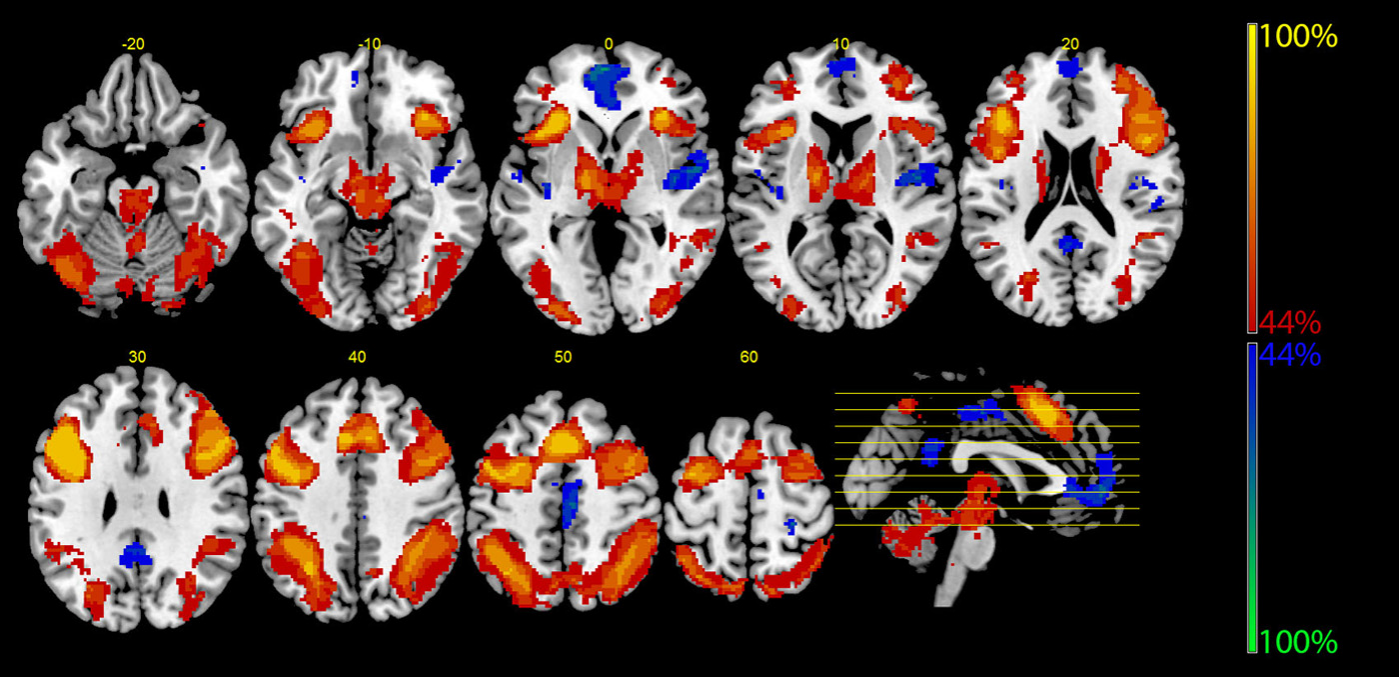
**The figure displays the probability maps for significant activations and deactivations across the nine studies shown in Figure 1**. Each study was binarized at a threshold of *p* < 0.001 and voxel-wise summed up. Thus, the displayed voxels reflect the probability (in %) that they were found to be activated (red) or deactivated (blue) by the nine studies shown in **Figure 1**. The red areas mainly reflect the EMN (see also **Figure 2**), while the blue areas correspond to the core areas of the DMN.

**Table 2** shows further details about the jointly activated areas in the conjunctions analysis, including hemisphere side, Brodmann area, *x,y,z* MNI co-ordinates, cluster voxel size, and threshold *z*-values. **Figure 3** shows statistical probability maps for % overlap for activated and de-activated areas across the nine Bergen studies. As can be seen in **Figure 3**, areas that are de-activated across the nine studies overlap with areas being active in the classic default mode network (DMN; Raichle et al., 2001), including ventromedial inferior frontal, posterior cingulate, parietal/precuneus areas. Thus, the probability maps seen in **Figure 3** reveal a negative relationship between the suggested generalized task-driven network and the DMN, such that activated and deactivated areas have non-overlapping spatial distributions.

**Table 2:** The table lists the anatomical structures, corresponding Brodmann areas (BA), peak voxel coordinates (MNI-space), corresponding *Z*-value of the most significant voxel within the cluster, as well as cluster size in number of voxel (2 mm × 2 mm × 2 mm).

Side	Anatomy	BA	*x*	*y*	*z*	*Z*(Conj)	Cluster size
Left	PCG, MFG, IFG	6, 44, 46, 47	–48	6	50	10,3	1999
Right	PCG, MFG, IFG	6, 44, 46, 47	48	32	36	10,0	3850
Left	IPL	7, 40	–30	–52	48	9,5	1519
Right	AG, MOG	19, 40	34	–72	30	8,5	880
Left	MOG	19	–26	–70	26	8,1	227
Left	SMA	32	–2	16	48	8,0	1220
Right	MFG	46	40	52	14	7,3	98
Left	ITG	37	–44	–52	–8	7,3	316
Left	MFG	46	–40	32	34	7,3	209
Right	PreCu	7	10	–68	50	7,3	154
Right	Thalamus		12	–10	2	7,0	556
Left	Caudate Nucleus		–10	–6	16	6,8	1154
Right	ITG	20	50	–48	–16	5,9	104
Left	MFG	46	–30	54	16	5,6	234
Right	MFG	10	32	54	0	5,2	24
Right	Hippocampus		26	–30	2	5,2	29

*PCG, PreCentral Gyrus; MFG, Middle Frontal Gyrus; IFG, Inferior Frontal Gyrus; IPL, Inferior Parietal Lobe; AG, Angular Gyrus; MOG, Middle Occipital Gyrus; SMA, Supplementary Motor Area; ITG, Inferior Temporal Gyrus; PreCu, Precuneus.*

## Activation Similarities to Tasks Which are Conceptually Unrelated

**FIGURE 4:**
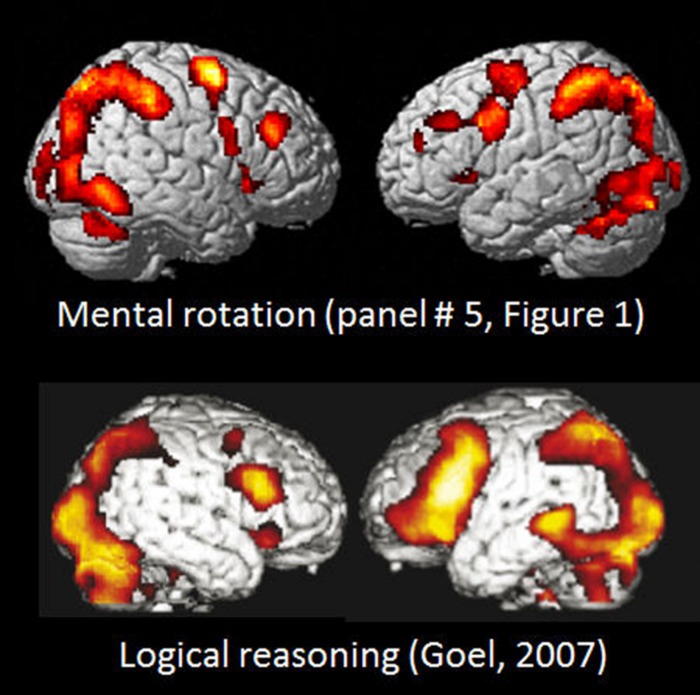
**Comparison of BOLD-fMRI activation patterns for Bergen Mental rotation task, and a logical deduction task (from Goel, 2007, Figure 1A).** Reprinted with permission from the Publisher.

A further example of the similarities in activation between tasks denoting apparent different cognitive processes is seen in **Figure 4** where the activation pattern for the left–right discrimination/mental rotation study (Hjelmervik et al., 2015) and shown in the mid-field panel (#5) in **Figure 1** is compared with the activation pattern obtained for logical deductive reasoning, and taken from Figure 1A from Goel (2007); originally from Goel (2003). The Goel (2007) article reviewed a series of imaging studies all concerned with activations to various aspects of logical reasoning, by exposing the subjects to various syllogistic and deductive reasoning tasks of the type “if *p* then *q*, *p*; therefore *q*” (see also Goel and Dolan, 2003; Prado and Noveck, 2007). It is apparent from **Figure 4** that the activations reported by Goel (2007) cannot be unique for logical reasoning, but are shared across tasks, since the extent and spatial distribution of the activations reported for a logical reasoning task for all practical purposes is identical to the activations that were reported by Hjelmervik et al. (2015) using a mental rotation/visual imagery task. This creates a challenge for imaging theory since a task requiring syllogistic reasoning is conceptually non-overlapping with a task that requires mental imagery and rotation of 3D objects.

## Extrinsic Mode Network (EMN) – A New Hypothesis

Instead the results from the nine studies in **Figure 1**, the conjunction analysis seen in **Figures 2** and **3**, and the close comparison of the two activation patterns seen in **Figure 4** below all point to the existence of a general cortical activation network that is shared across cognitive tasks, themes and domains. We have called this network a general purpose *extrinsic mode network* (EMN). The EMN is similar to what Hugdahl et al. (2012) labeled the “effort mode network” when describing a common activation pattern across different cognitive tasks and paradigms. We have, however, chosen to re-label the EMN as the EMN since it avoids any association with mental effort and task difficulty, which is not what the EMN is suggested to reflect.

A first impression of the activation patterns seen in **Figures 1–3** is how different cognitive tasks and stimuli could produce such overlap in activations, that the tasks produced similar overall neuronal networks independent of the cognitive process that was induced. However, if the activation patterns shown in **Figures 1–3** are not unique for the cognitive processes that were explored in the first place in the respective studies, but rather reflect an underlying commonality of a generalized network that responds to the allocation of non-specific cognitive resources, independent of the specific nature of the task then there was nothing wrong with the activations seen in **Figure 1**.

The notion of the EMN extends the concept by Duncan (2013) who proposed a multiple demand system (MD), and which he suggested is a common activation network across cognitive tasks and dimensions (see also Fedorenko et al., 2013; Cole et al., 2014). Other suggestions have been fronto-parietal control system (Vincent et al., 2007), superordinate cognitive control network (Niendam et al., 2012), task-related network (Fox et al., 2005a, 2009), task control network (Dosenbach et al., 2007), or dorsal and ventral attention systems (Fox et al., 2006), task-general network (Cole et al., 2014). We suggest the EMN as an umbrella term for all these networks that share a common activation pattern structure, and being up-regulated during task processing, but independent of the specific cognitive task-structure.

## The Underlying Mechanisms – Is Attention Special?

**FIGURE 5:**
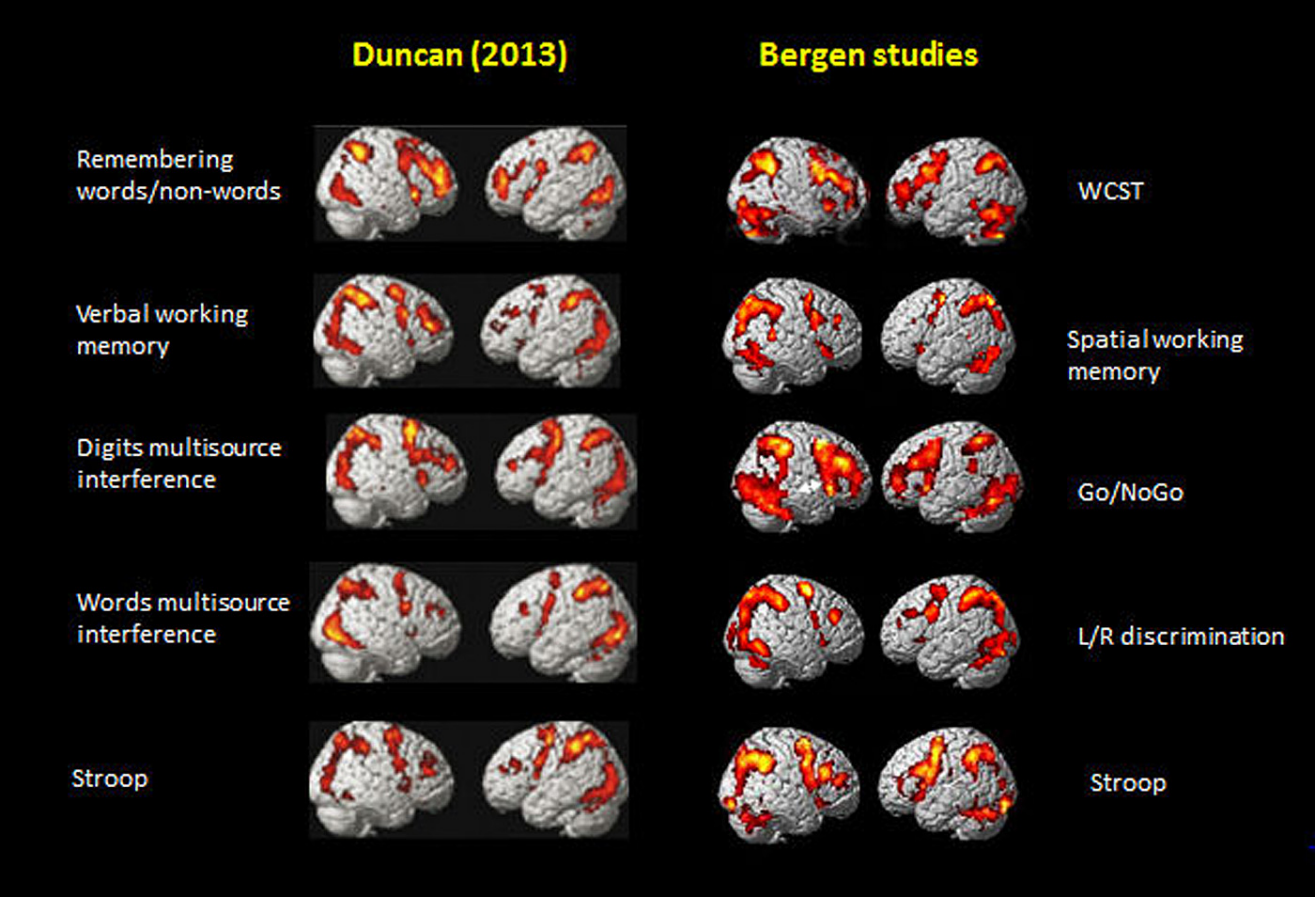
**Comparison of activation patterns between five of the tasks shown in Figure 2 in Duncan (2013).** Reprinted with permission from the Publisher, and selected five tasks from the Bergen series of studies shown in **Figure 1** of the current paper.

**Figure 5** shows the activations rendered on a standard SPM template for five of the seven tasks from Figure 2 in Duncan (2013) and corresponding activations from five selected tasks from the Bergen studies (panels #1,2,3,5,6, in **Figure 1**), Again, the similarities in the overall pattern of activation seen in **Figure 5** between the two sets of tasks from the Duncan (2013) article and the Bergen studies is striking considering that almost everything that one could conceive of being different between the two studies, also is different, including, tasks, and when cognitive processes overlap, task-specific parameters nevertheless differ, subjects, MR scanners, analysis software and statistics, etc. The fact that the resulting activation patterns for the two set of tasks is almost identical even from a simple ocular inspection, despite the between-comparison “noise” in the data, speaks to the robustness of the EMN.

Duncan (2013) restricted, however, the significance of the MD to be specifically responsive to fluctuations of attention episodes and epochs across time. Similarly, Fox et al. (2005a, 2009) suggested that the task-positive activations they observed when studying the DMN were related to attention and goal-directed behavior as a general principle of cognitive functioning. The EMN shares properties with what Fox et al. (2005a) discussed as goal-directed task network which govern much of our existence, i.e., a task-positive network consisting of the dorsal attention network along with a constellation of areas that have included a fronto-parietal control network and a salience network. As argued above, however, these different networks are not unique from a cognitive point of view since they overlap despite being elicited by different stimuli and situations. Duncan (2013) referred to the MD system as reflecting “attentional episodes” which could be understood as the floating of attention from one focus to another across time, as a kind of glue that makes us aware of aspects of the surrounding reality.

### Focused Awareness and the EMN

We prefer another term for underlying mechanisms, namely episodes of extrinsically directed “focused awareness” because it does not require the specification of an additional term, attention, as an operationalization for the same underlying neuronal processes. In that sense, the EMN is like what Norman and Shallice (1980) labeled a “supervisory attentional system,” as a superordinate system that would activate other cognitive systems and processes under appropriate conditions. The supervisory attention system would activate subordinate cognitive schemas with different probabilities depending on the impending cognitive context, and would apply general cognitive strategies for monitoring and supervising appropriate actions. The supervisory attention system resembles aspects of the EMN. The system proposed by Norman and Shallice (1980) is, however, a purely cognitive system, with no specific reference to the underlying neuronal architecture. It moreover puts attention at the core of cognitive activity for allocating the necessary cognitive resources for appropriate action. As we have argued above, attention cannot be at the core of such strategic planning and executive functioning (cf. Duncan, 2013), and we therefore suggests that the EMN transcends attention, which instead is seen as a subordinate cognitive process that is guided by the EMN, not the other way around.

The EMN is also similar in spatial anatomy to what Corbetta et al. (2008) called the dorsal attention and cognitive control networks, which are activations that show spatial coherence across time between a seed region of interest and other brain areas (see also Biswal et al., 1995; Greicius et al., 2003; Raichle, 2010). This network has been shown in task-related fMRI studies (Corbetta et al., 2008), and as patterns of spatial connectivity obtained during resting state conditions which are observed during periods of absence of sensory stimuli and active task-processing (cf. also Zhang et al., 2008; Smith et al., 2009). Further, Corbetta et al. (2008) in addition also defined a ventral attention network, important for interrupting the dorsal network and thus reorienting attentional focus. This mainly right-lateralized network anatomically overlaps with the more bilateral dorsal attention network within the right middle frontal gyrus, and has been observed also in both task related fMRI and resting-state fMRI. Due to their presence also at rest, these additional networks are therefore part of the brain’s intrinsic activity, i.e., the DMN (Raichle, 2010, 2015a).

### Intrinsic and Extrinsic Brain Activity

With intrinsic activity we mean a marker of the brain’s ongoing activity in the absence of a specific task or stimulus, i.e., a marker of non-specific neuronal activity (cf. Cole et al., 2014). Interestingly, the dorsal attention network and the brain’s DMN seem to broadly act in an anti-correlated way (but see also Popa et al., 2009; Foster et al., 2015), while the ventral network acts independently and is suppressed during episodes of focused attention. As illustrated above, the EMN is active under all conditions where participants are asked to take initiative, like responding to a certain stimulus, selecting an appropriate stimulus among distractors, or process novel information, but the activation of the EMN appears to be independent from the actual content of the stimulus and the stimulus modality. The EMN is therefore a marker of the brain’s extrinsic activity. Moreover, activations that depend on stimulus content and stimulus modality are particularly seen outside of the EMN. For example, tasks involving spatial processing often show increased activity in areas, adjacent to the EMN within the right prefrontal cortex, while verbal stimulus material activates in particular left prefrontal areas (cf. Lie et al., 2006; Lycke et al., 2008).

## The EMN is not Just Response to Task Difficulty

A question remains, namely whether task difficulty modulates the EMN in terms of activation strength or areas involved. Looking again across the different studies (**Figure 1**), the pattern appears to be quite stable, irrespective of variations in task difficulty and mental effort involved in the different studies. Thus, the EMN should not simply be responding to increased task difficulty, which moreover should be task-specific, while the EMN is task non-specific. Further, the EMN broadly overlaps with the network for intrinsic alertness, which is the most basic level of attention (Sturm et al., 1999), indicating that it is active also at a very basic level, which may indicate that it is always activated when a possible response is required or expected. Unpublished data from Specht et al. (2009) support this notion by showing that already presenting an instruction to the subject creates an activation pattern that shares a remarkable similarity with the EMN. However, at the other end of the spectrum of task complexity, Lie et al. (2006) and Specht et al. (2009) explored the neuronal correlates of a task that requires cognitive processes like parallel processing of several stimulus features, working memory, hypothesis generation, and response adaptation. Although the intensity of the EMN activity varied to a certain degree with tasks demands, the overall pattern of the EMN remained remarkably constant in the two studies. The comparison between Lie et al. (2006) and Specht et al. (2009) studies reiterate the described observation that the activation within the left prefrontal cortex is mainly stimulus dependent, while the activation of the right prefrontal cortex, as part of the EMN, is not.

### Non-Specific Cognitive Resources and Net Balance of Extrinsic to Intrinsic Activity

A common characteristic across all tasks reviewed so far is that they require allocation of non-specific cognitive resources, irrespective of whether this is for solving cognitive conflict, resolve a difficult perceptual task, store items in the working memory buffer, repeatedly shift attention focus, attain goal settings, set up cognitive plans or processing strategies, etc. In this respect, the EMN and DMN modes would act as neuronal correlates of what Kahneman (2011) called the “fast” and “slow” modes of thinking, with a fast mode of thinking being automatic, overlearned, and non-reflective, and corresponding with the DMN, while a slow mode of thinking is corresponding to the EMN network.

A way of conceptualizing the relationship between the EMN and DMN would be to think of the EMN as an extrinsic mode network, and the DMN as an intrinsic mode network, and that the activation recorded at any point in time correspondingly reflects the net balance of extrinsic to intrinsic activity across time. All the instances listed above could collectively be labeled higher cognition, and they have a common feature that cannot be successfully managed without the allocation of non-specific cognitive resources. Thus, we suggest that the activation network, the EMN, or MD system in Duncan’s (2013) terminology, which is associated with all these tasks and processes, correspondingly reflects allocation of non-specific cognitive resources at the neuronal systems and circuitry level of explanation.

## The Operational Nature of Cognitive Processes and Functions

Even if it is accepted that the human brain up-regulates a cortical network that contains nodes, or regions, mainly in the frontal and parietal lobes that connect during task processing to constitute a network (Fox et al., 2005a, 2009; Vincent et al., 2007; Niendam et al., 2012; Duncan, 2013; Fedorenko et al., 2013; Cole et al., 2014), what is not clear is, however, the nature of the cognitive tasks that elicit this network. It has previously been suggested that, besides basic aspects of attention like intrinsic alertness (Sturm et al., 1999), it responds to executive control, focused attention, goal maintenance, strategy selection, and performance monitoring (Fedorenko et al., 2013), attentional episodes (Duncan, 2013), and response conflict, novelty, and overcoming a pre-potent response tendency, working memory, perceptual difficulty (Duncan and Owen, 2000). A problem with conceptualizing cognitive processes is that they are mainly defined operationally, where the underlying conceptual identity is not known. For example, the name “executive” is just a metaphor taken into neuropsychology from the business world, denoting a process like the executive director who sits at the top of the organization hierarchy and controls the planning and decisions of the company. Similarly, the term “working” in working memory is a metaphor for the part of memory which is active, i.e., “working” at any given time. The point we would like to make is not that these metaphors necessarily are wrong but that they do not have clear definitions and identification of the underlying cognitive processes that they denote, and which goes beyond the operational level of explanation. In this context, the term “executive” lacks substance and should probably be replaced by terms that are more easily operationally defined. Therefore, the existence of a common task-related network across cognitive domains and processes, which are processes that at best are only operationally defined, should not come as an unreasonable suggestion. In the absence of knowledge of what the core underlying mechanisms are for cognitive tasks such as; executive, working memory, attention, cognitive control, perceptual difficulty, goal planning, decision making, etc., we now suggest that a common denominator for these tasks is that they all require the allocation of task non-specific cognitive resources and extrinsically directed behavior, which mainly engages frontal and parietal areas, primarily on the lateral surface of the brain, and being right lateralized in the prefrontal cortex and bilateral in the parietal lobe.

## Task-Characteristics of the EMN

There are some notable characteristics of the EMN, aside the commonalities in frontal and parietal activations across tasks and processes (see **Figure 1**). One characteristic is the apparent asymmetry between the hemispheres, with the right prefrontal cortex activation typically showing a quartet of nodes, with three nodes along the precentral sulcus, and a fourth node located anterior to the central sulcus and the frontal eye fields. The most superior activation of this quartet is typically seen close to the frontal eye field. Inferior to it, a second activation occurs, typically seen at the intersection of the middle and inferior frontal gyrus, and the most inferior activation is within the ventro-lateral prefrontal cortex. The fourth activation is typically seen more anterior, within the middle frontal gyrus. These four nodes occur independently of the task and appear as a general network. In contrast, the left hemisphere demonstrates a task dependent variability in activations, as described above. Thus, different levels of asymmetry emerge within the prefrontal cortex, depending on the task demands and the content of the task.

## Absence of Verbal Tasks – Is Language Special?

Another characteristic is that the tasks that so far have been shown to elicit the EMN, and shared with the tasks used in the studies by Duncan and Owen (2000) and Fedorenko et al. (2013) are primarily visual non-language tasks. This was also noted by Fedorenko et al. (2013), who observed that the regions implicated in a corresponding EMN-like network are mainly driven by visuo-spatial tasks rather than by language-tasks, and that level of difficulty in solving the task does not apply to the typical language networks. Moreover, the EMN appears to surround language related areas (Duncan, 2013) and does not overlap with them; particularly it does not overlap in the left prefrontal cortex. Further support for this comes from a meta-analysis by Indefrey and Levelt (2004), who explored areas for word production and found areas, mainly covering left frontal areas but spare a couple of core areas of the EMN within the right frontal and parietal lobe. This observation is an another evidence that overlearned tasks – and language clearly is overlearned and seemingly effortless – do not activate EMN, with the possible exception of an unfamiliar, second language.

We have made the same observation in the Bergen studies, also seen in the tasks that we have data from, and shown in **Figure 1** and **Table 1**. Of these tasks, seven were visual, while two were auditory tasks task (Eichele et al., 2005; Falkenberg et al., 2011). The task used by Falkenberg et al. (2011) was also a verbal task, and it may be worthwhile noticing that this task is the only one of the nine tasks listed in **Table 1** that activated the posterior temporal lobe, particularly on the left side, in the vicinity of the peri-Sylvian region, and overlapping with both the primary auditory cortex and the language areas. The same areas have previously been implicated in speech perception and auditory processing (Binder and Price, 2001; van den Noort et al., 2008; Price, 2012; Specht, 2014). An exception to the non-language aspect of the EMN would be the Stroop task, which is also a verbal task. However, the condition that elicits the EMN is where the subject is instructed to report the ink-color the word is written in, suppressing the language/semantic aspects of the stimulus. As mentioned above, the EMN is typically observed in visual tasks, and less frequently in language tasks, which may indicate that language is overlearned in general. However, the reason that EMN is observed in the Stroop task arises from the inhibition of an overlearned response tendency to process the semantic component of the incongruent color–words (Brass et al., 2005), which requires a certain degree of non-specific cognitive resources and thus trigger the EMN.

## Task-Novelty and Cognitive Challenges

The tasks used in the nine Bergen studies shown in **Figure 1** all require the solution to a novel challenge where the subject has to provide an answer, which could be right or wrong. Examples from **Figure 1** of such tasks are; Stroop, *n*-back Working memory, Go/NoGo, Left/Right confusion, and mental rotation, WCST test. In all these instances, the subject has to go through a series of mental operations in order to provide a solution to the challenge or problem exposed to him/her, and in such instances the EMN is up-regulated to provide the background resources necessary for being able to solve the task. The specifics of each task will then require resource allocation, such as searching the mental lexicon, perceptual transformations in 3D space, attentional filtering, stimulus inhibition, and suppression, etc. Each of these sub-processes will require additional cognitive resources, which will have their neuronal footprints, but they all share a common cognitive and neuronal infrastructure of non-specificity in order to solve the task, and provide a correct answer. Other terms that would fit the notion of a task non-specific EMN in order to meet requirements for task solving would be mental flexibility and cognitive plasticity (cf. e.g., Blumstein and Amso, 2013). An additional characteristic of the EMN is that it should be sensitive to experience and learning, which by definition also reduces novelty. In this sense, the EMN is hypothesized as a dynamic, and not static, network that may show individual differences in core EMN activations between individuals to the same tasks, depending on the amount of previous experiences, and thus reduction of novelty. Using again the picture by Kahneman (2011), learning would imply a gradual shift from a “slow” to a “fast” mode of thinking and thus from EMN to DMN. In turn, inhibition of (over-)learned actions through adaptation of responses to a novel situation would cause a rebound to the “slow” mode of thinking, indicating a higher level of non-specific cognitive demands, and thus increasing the probability of activation of the EMN network.

## Different Cognitive Tasks, Similar Activations – Not a New Idea

The idea that different cognitive tasks and processes engage the same or similar neuronal regions is not new. This was to our knowledge originally suggested by Duncan and Owen (2000) and these authors seemed surprised by their observatio*n; “The results show a striking regularity: for many demands, there is a similar recruitment of mid-dorsolateral, midventrolateral and dorsal ACC. Much of the remainder of frontal cortex, including most of the medial and orbital surfaces, is largely insensitive to these demands.” (p. 475).* A somewhat similar idea was expressed by Køhler et al. (1998) who suggested the existence of domain-specific vs. domain-general brain structures involved in semantic memory, see also Cabeza and Nyberg (2000) and the need for generalized brain structures in order to handle requirements for mental flexibility. Perhaps as a consequence of the ruling Zeitgeist at the time, Duncan and Owen (2000) only considered that their observations showed evidence for regional specialization of function within the prefrontal cortex, i.e., that the activation across tasks and processes was region-specific, and not part of a network. The network idea was, however, present 13 years later in the paper by Duncan (2013; see also Duncan, 2010) where he suggested that the activation regularities across tasks constituted a multiple-demand (MD) system that has all the qualities and characteristics of a cortical network; “*Taken together, these data show tightly localized MD activity, varying in exact pattern from one person to another but with a highly consistent overall topography in frontal and parietal cortex.”* (p. 37). Similarly, Fedorenko et al. (2013) writes; *Comprising this network are regions on the dorsolateral surface of the frontal lobes (along the inferior frontal sulcus/middle frontal gyrus), parts of the insular cortex, regions along the precentral gyrus, preSMA, and SMA), parts of the anterior/mid cingulate, and regions in and around the intraparietal sulcus. We will refer to these regions as the MD system* (p.16616).

## Comparison with the Fedorenko et al. (2013) Study

We have taken Figure 6 from Fedorenko et al. (2013), which shows the averaged activation across the seven different cognitive tasks they used (cf. also Duncan, 2013), and displayed it together with one of the Bergen studies (Stroop *n*-back task; data from Griffiths et al., 2013), visualizing both lateral and medial activations. Fedorenko et al. (2013) compared activation patterns for seven different tasks that included semantic memory, number arithmetic, spatial and verbal working memory, Stroop task, and two multisource interference tasks, that required inhibiting one source of stimuli in order to process another source.

**FIGURE 6:**
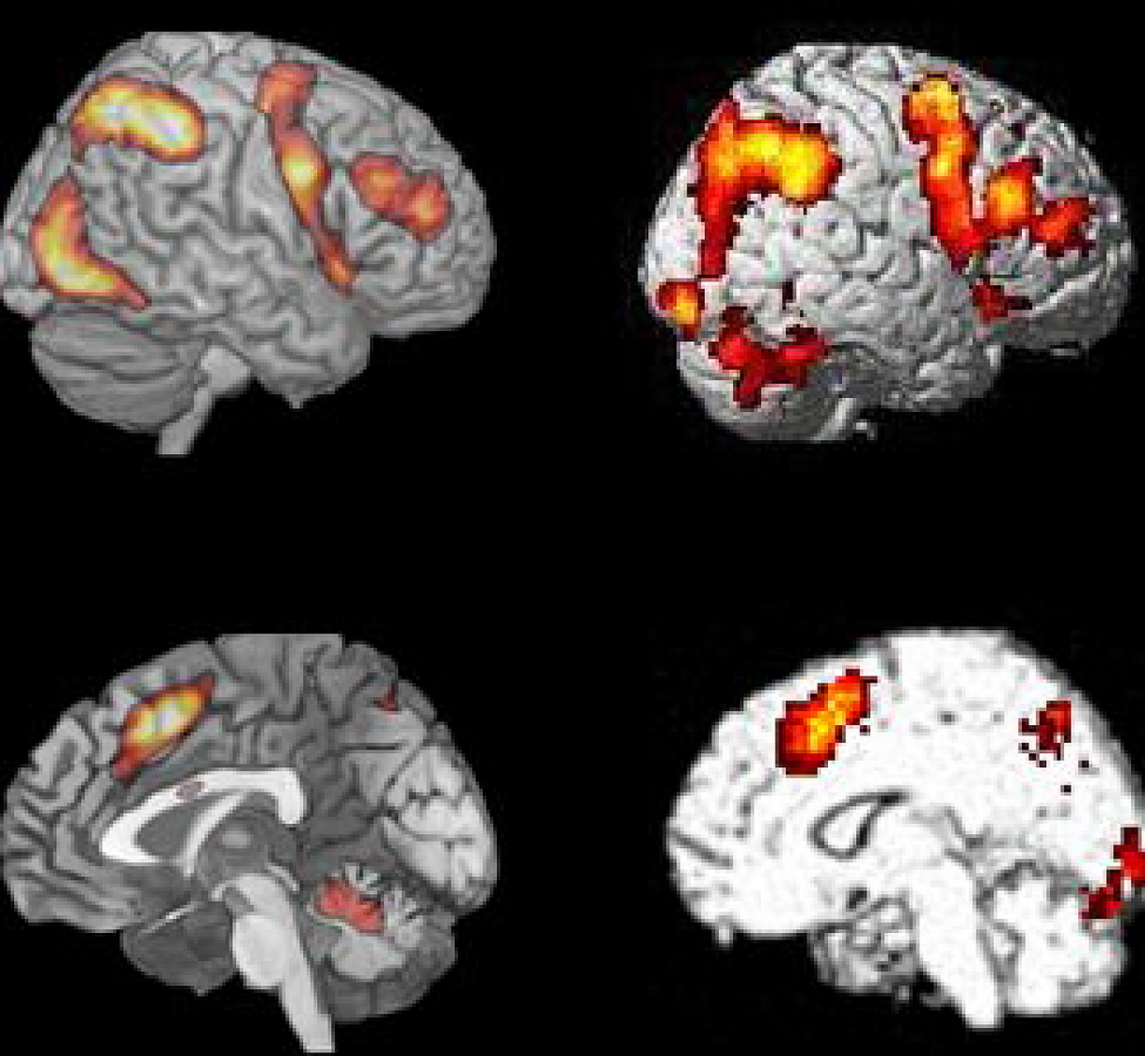
**Comparison of BOLD-fMRI for the Bergen Stroop task (panel #5 in **Figure 1**), and the average activation shown for the seven cognitive tasks used by Fedorenko et al. (2013) Figure 2.** Reprinted with permission from the Publisher.

The similarities in **Figure 6** are again both striking and remarkable considering the range of cognitive processes included in the studies in the comparison. Fedorenko et al. (2013) made the argument that previous group-analyses which have shown commonalities of activation across tasks may have overestimated their case because of inter-individual variability in brain anatomy, which could result in overlapping activations at the group level despite that individual activations are different. This argument could, however, be discussed since activation data typically are normalized to a standard template before visualization in order to avoid the kind of confounding that Fedorenko et al. (2013) warn against. What they did not comment on, however, is that running the same subjects on different tasks, likewise may overestimate overlapping activations, because these same subjects may be non-representative for the population at large, a potential problem that will increase in strength with decreasing sample sizes. Since Fedorenko et al. (2013) based their critical analyses on only 12 subjects this problem is not trivial. A solution would be to have different subjects run on different tasks, and then look for commonalities in the activation patterns. This was done in the studies shown in **Figure 1**, with 187 subjects in total. It is therefore interesting to note the close overlap between the activation patterns shown in **Figure 1** and the aggregate activation pattern shown by Fedorenko et al. (2013, see **Figure 6**).

## Intrinsic vs. Extrinsic Activity, and the DMN and EMN Networks

Ever since the discovery of the DMN by Raichle et al. (2001; see also Shulman et al., 1997; and recently reviewed by Raichle, 2015a,b), it has been assumed that the brain, in the absence of any external stimuli or instruction, has a unique activation architecture, whereas external stimuli or instructions will drive task-specific activations which are anti-correlated with the DMN (Fox et al., 2005a, 2009). The DMN is often described as the “resting-state” network although that may not be an appropriate description since the DMN is only one of many identified cortical networks during resting (Smith et al., 2009). The term “default mode” is better seen as a name for the brain’s default, or base-line state under certain conditions, and the DMN has also been described as the default mode of *intrinsic* neuronal activity (Fox et al., 2007; Raichle, 2010, 2015a,b). We now suggest that the EMN acts like base-line activation during states of active task-related processing and further suggest that the EMN represents a basic network for *extrinsic* neuronal activity. With extrinsic neuronal activity we mean the brain’s activity to specific stimuli or tasks, in contrast to intrinsic neuronal activity which is the brain’s activity in the absence of specific stimuli or tasks.

While the existence of the intrinsic DMN is empirically corroborated through the work of Raichle and colleagues (see Raichle et al., 2001; see also Binder, 2012 and Buckner, 2012 for the history of a resting-state network), the EMN is predicted to exist for the reasons given in the beginning of this article. Empirical evidence for the existence of anti-correlated task-negative and task-positive networks have primarily come from the studies by Fox et al. (2005a, 2009), but also from others. These studies should, however, be considered as empirical confirmations under standard resting state conditions, since the data for the analyses have been obtained during resting state conditions, using seed-voxel correlational approaches. We now suggest that the DMN and EMN show a similar inverse relationship, obtained under both task-absence and task-presence conditions, and not attributable to global signal fluctuations during resting state (task absence) conditions (cf. Fox et al., 2009).

## Network Generality vs. Specificity – Where are the Limits?

An issue not discussed so far is the question of generality versus specificity of a network for extrinsic activity, i.e., the EMN. The generality issue relates to how similar the network is across tasks, and the specificity issue relates to how the network is modulated by the specifics of the task, and the underlying idiosyncratic cognitive processes. A network consists of nodes and edges, thus contrary to the general mythology in the imaging community, a network approach does not replace a so called “blobology” approach, it just connects the “blobs,” which in graph theory terminology are called nodes, or vertices. Nodes are connected through edges, or arcs, and together nodes and edges are the elements of a network. Nodes can be of two different types, satellites and hubs, where hubs are strongly connected nodes, and satellites are correspondingly weakly connected nodes. The strength of connectivity in a network is measured as the degree, which for undirected edges is the sum of all edges connecting to the node, and for directed edges is the sum of all weights attached to the edges connecting to a node. An important parameter for a graph-theoretic description of a network is the shortest path length, which describes the minimal number of nodes one has to pass for moving from one part of the network to another, also known as the distance matrix. In general, the graph theory provides a broad range of various measures for describing the inherent complexity of a network structure, its information flow, and its capability to compensate perturbations (Sporns, 2012).

Without going much into details on the different parameters, we suggest that EMN-hubs in the frontal and parietal lobes are invariant and task non-specific, while shortest path length and degree of connectivity between activations are task variant and may change depending on the specifics of the task, like stimulus content or task demands. In other words, although the overall hub structure of the EMN will be the same for a working memory and mental rotation task, the graph-theoretic description of the network may vary. Although there is an emerging interesting in descriptions of network complexity and connectivity patterns of resting state networks (Friston et al., 2014), like the connectome (Sporns et al., 2005), there is, however, to our knowledge, no in depth graph-theoretic description of the network, underlying the EMN.

## EMN Network Nodes and Hubs

We would like to make the argument that the EMN nodes are areas not necessarily shared by all tasks, while the hubs are activated areas that are shared for all tasks requiring above threshold allocation of non-specific cognitive resources. In the studies reviewed so far we suggest that key hubs in the EMN are the SMA, pre-central sulcus, inferior frontal gyrus and the insula, and inferior parietal lobule. The dynamics of the EMN would also mean that the peak amplitude and/or area extension of the “blobs” will vary across tasks, although not affecting the overall architecture of the whole network (c.f. Lie et al., 2006). In this sense, a EMN analysis approach can accommodate both network generality and network specificity as discussed above.

## EMN and DMN Activation Loci

The DMN (Shulman et al., 1997; Raichle et al., 2001; Buckner et al., 2008; Raichle, 2010) is an activation network observed during periods of non-task activity, i.e., during periods of cognitive non-effort, sometimes called periods of “mind-wandering” (Christoff et al., 2009; Smallwood et al., 2013, but see however, Raichle, 2015b). The DMN has an activation configuration that includes the medial temporal lobe, ventromedial frontal lobe, posterior cingulate cortex, precuneus, and lateral inferior parietal lobule, i.e., a different configuration than the EMN. The differences in activation foci between the two networks can be summarized such that the DMN shows activations toward medial and posterior regions, while the EMN shows activations toward lateral and anterior regions, but also with overlapping activations in frontal and parietal areas (Eichele et al., 2008; Løberg et al., 2012; Nygård et al., 2012).

## Network Dynamics and Interactions – Implications for Mental Disorders

Manoliu et al. (2014) found aberrant regulations of the DMN in schizophrenia and what they called a central executive network during episodes of resting state with no demands for task-processing. Aberrant DMN activation in schizophrenia has been found in several other studies and could be seen as an established consensus in the field (e.g., Williamson, 2007; Mannell et al., 2010; Rotarska-Jagiela et al., 2010; Nygård et al., 2012; Razavi et al., 2013; Baker et al., 2014). In addition, van Lutterveld et al. (2014) found increased DMN activation in key brain areas in non-psychotic individuals that are prone for experiencing hallucinations, pointing to aberrant DMN activation may be a trait property for hallucinatory experiences. This is an interesting finding considering that other studies have repeatedly observed similar results in hallucinating psychotic patients (e.g., Jardri et al., 2013; see also Aleman and Vercammen, 2012 for review of functional connectivity and hallucinations). Of particular interest for the current study is the hypothesis put forward by Northoff and Qin (2011) that hallucinations in schizophrenia may be a special condition caused by aberrant resting state activation in these patients, in their own words; “⋯*based on recent findings, we therefore developed what we call the ‘resting state hypotheses’ of AVH. Our hypothesis suggest that AVH may be traced back to abnormally elevated resting state activity in auditory cortex itself, abnormal modulation of the auditory cortex by anterior cortical midline regions as part of the DMN, and neural confusion between auditory cortical resting state changes and stimulus-induced activity.”* (p. 202). Hugdahl et al. (2009a,b) suggested that the cognitive impairment and hypo-activation seen in, e.g., schizophrenia patients when exposed to challenging cognitive tasks could be caused by failure of interactive regulation of the DMN and EMN networks, rather than a deficit with regard to a specific brain region. Hugdahl et al. (2009a) wrote that; “*it is proposed that cognitive impairments in schizophrenia, including inhibitory control of hallucinations⋯, may involve failure of down-regulation of a resting-state network and corresponding up-regulation of an effort network, thus upsetting the normal functioning of cognitive control mechanisms.”* (p.40). They further continued; “*a generalized effort network would be activated whenever demands for recruitment of higher-order cognitive functions, like attention, working memory and executive, or control functions, are called for and would show activations in prefrontal cortex, anterior cingulate, and inferior parietal cortex. A generalized effort network would be activated orthogonal to the default mode or resting state network as suggested by* Fox and Raichle (2007).” (p. 41). A network dynamics perspective would also be valid for understanding of other mental disorders, such as bipolar disorder, where the switching between hyper- and hypo-activated mood states is a core symptom, and where the underlying neuronal mechanisms are not known. Aberrant resting state activation was observed in bipolar disorder by Calhoun et al. (2012) who found that bipolar patients showed different resting state activation than schizophrenia patients. This activity was, however, not compared to task-processing activation in the two groups, thus, whether schizophrenia and bipolar disorders differ in network dynamics in a more general sense is not known. Kindler et al. (2015) argued that in order for any goal-directed activity to be up-regulated, default mode activity has to be terminated, and that schizophrenia patients show increased sustained activation in the posterior parts of the DMN. This is similar to the observation of differences in onset and offset transients, respectively, reported in the earlier work by Raichle and colleagues (e.g., Lustig et al., 2003; Fox et al., 2005b), which would consequently suggest interference with the up-regulation of the EMN.

## Up- and Down-Regulation Dynamics

We have so far conceptualized the interaction of the DMN and EMN as interference, in the sense that when one is up-regulated the other needs to be down-regulated. However, the interaction is probably best described as of a dimensional interaction, such that the up-regulation of the EMN is not dependent on the full down-regulation of the DMN, but that the relationship may follow a quantitative, correlational, trajectory. This would be similar to what Popa et al. (2009) found for the relationship between the DMN and task-related activity, and also for the relationship with sleep and anesthesia which demonstrated a gradual, rather than categorical relationship between resting-state and task-related activity (cf. Greicius et al., 2008; Foster et al., 2015). We further suggest that the EMN follows a threshold effect, which will vary depending on task novelty and which would be predicted to be inversely related to DMN down-regulation. When task novelty is high, and correspondingly overlearning is low, the EMN will be elicited even when the DMN is not fully down-regulated. Conversely, when task novelty is low, and correspondingly overlearning is high, the elicitation threshold for the EMN will be higher, i.e., the DMN must be down-regulated to a higher degree. These predictions can be empirically tested by designing tasks that will vary in novelty and previous learning experiences at the individual level.

## Switching between Resting and Task Processing Periods in Everyday Life

There are two issues that need to be resolved in order to best study the interference effects of DMN and EMN activity. The first is that the DMN is typically acquired during prolonged scanning periods of between 10 and 30 min of resting in silence, except for scanner noise, allowing for the mind to “wander freely” (Gruberger et al., 2011; Sood and Jones, 2013 for reviews). Brain activation data acquired during such periods are then for example compared between a clinical and healthy control group, and typically finding that the clinical group show aberrant activation compared to the control group. It is then concluded that DMN hyper-activation causes interference with task-processing in the clinical group, which would then explain why, e.g., schizophrenia or depressed patients show impaired cognitive functioning. The problem with such a conclusion is that the subjects are typically studied only during a resting period and not also during a task-processing period (see however, Whitfield-Gabrieli et al., 2009; Mannell et al., 2010), which makes it difficult to draw conclusions about how the same groups would perform during active task-processing. Several studies have shown that psychotic patients reveal disruption of cortical connectivity during resting periods, even with large samples (e.g., Baker et al., 2014). However, these studies typically scan patients and controls only during resting, it is therefore not possible to conclude whether the same patients also show disruption of EMN, which would require an analysis across different tasks, or an analysis of up- and down-regulations of the DMN and EMN during alternations of task-absent and task-present epochs in the course of the MR scanning session. A similar problem will appear when different methods are used for analyzing resting-state data. The resulting component “blobs” and hence the inferred network connectivity from different analysis approaches may vary across analysis domains, such as intensity, spatial extension, and even what may constitute an activation (“blob”). It has been shown that there is competition between resting state and task processing periods that correlates with fluctuations in default mode activation (Greicius and Menon, 2004), and that spontaneous lapses in attention in between focused attention epochs also modulates the strength of default mode activation (Weissman et al., 2006; Eichele et al., 2008). It therefore seems reasonable to assume that there should be ongoing up- and down-regulations of resting and task processing activity during the course of a day, which cannot be concluded from monitoring network activity during resting periods, or between separate resting and task-processing periods.

The second problem is that prolonged rest periods, as in the typical MR scanning situation, in the absence of any competing stimuli is an artificial situation, seldom encountered in everyday-life, where such experiences are constantly interrupted by interfering stimulus-processing episodes. Also problematic is the notion of the DMN as a marker of “mind-wandering” (Christoff et al., 2009) or “day-dreaming” (Kucyi and Davis, 2014). As pointed out by Raichle (2015b), the fact that the DMN is present under anesthesia in both humans and other animals, and in sleep (Fukunaga et al., 2006) suggest that the activation seen in the DMN during resting-state periods is not necessarily restricted to unconstrained conscious experiences, like mind-wandering. A better situation would perhaps be listening to music as a kind of mental resting (which interestingly is a situation where an external stimulus is present, but with no task-requirements). The only study we know of that has investigated DMN and music experience is Kay et al. (2012) who found that music listening was a valid condition under which the DMN could be studied.

However, the examples given above are exceptions, and the normal every-day experience of most people is not of lying on the beach day-dreaming, or listening to music, most of the day, doing nothing, but rather going in and out of task processing and periods of rest. Thus, a key question is how to operationalize the switching in and out of the two network modes in the scanner situation. This would better model the everyday-life situation, with shifting demands in between brief mind-wandering periods (see Shulman et al., 1997 for an example of such analysis).

## Outstanding Questions

There are several outstanding questions with regard to the conditions under which a generalized mode network for extrinsic activity will be elicited. Here we list some of these questions that should be sorted out in future research:

• The relationship between the DMN and EMN is probably not absolute in the sense that when one is present the other is absent, but the exact nature of the relationship is not known. In other words, to which degree is one network less expressed than the other in an interaction between the two, and is there a third region or network that mediates this interplay.
• Is the EMN dependent on one or several key cognitive sub-processes which act as eliciting factors? Although discarded as a unique elicitor in the discussion above, attention may still exert a key role, such that a task with low attention load would have less power to elicit the EMN, independent of the difference in cognitive demands across tasks.
• Are the underlying physiological mechanisms (receptors, transmitters) same or different for the EMN and single process-driven activation? The increasing use of MR spectroscopy together with BOLD-fMRI recordings will allow for quantification and correlations of levels of brain metabolites and transmitters, like glutamate, glutamine, GABA, choline, creatine, NAA and others, with BOLD data. In this way it would be possible to relate specific transmitters to the two networks.
• Are the EMN and DMN networks actually two sides of a single larger brain system? A recent study by Foster et al. (2015) may provide a first hint that task-positive activation patterns may be components of common, higher-order network interactions. Foster et al. (2015) used intracranial EEG recordings from the cortical surface and found that high-frequency gamma band equivalents of DMN components were present not only during resting and sleep, but also during active task-processing. Popa et al. (2009) reported similar results, when recording unit activity and local field potentials from the cat cortex. Popa et al. (2009) found evidence for what they called “large-scale coordination of activity” in parieto-temporal and cingulate cortex regions, respectively, that were associated with task-positive and task-negative stimulus conditions. Since these connectivity-overlaps often are hidden in the neuronal noise produced during these states, overlapping epochs of the activation patterns gets buried in the background noise, while anti-correlated epochs stand out, and are therefore more easily seen. Our previous thinking might therefore have been misled by the fact that these networks at certain points in time are anti-correlated, making us assume that they must be separate networks, rather than components of one single system.

Hopefully, future research will resolve these issues and questions, and also apply an EMN/DMN perspective when studying aberrant network connectivity and network interaction dynamics in clinical groups, which can open up new avenues for the understanding of the neuronal underpinnings of some of the most severe mental disorders. A network dynamics approach may also apply to the study of other mental states, than a mind wandering resting state versus active task-processing. As mentioned above, the mental state induced by listening to music is one such state, where the neuronal substrates are largely unknown (see however, Blood et al., 1999; Zatorre, 2003). It should also be of interest to use a network dynamics approach with consecutive up- and down-regulations of the EMN and DMN across time in patients with mental and neurological disorders and diseases.

## Conflict of Interest Statement

The authors declare that the research was conducted in the absence of any commercial or financial relationships that could be construed as a potential conflict of interest.
